# A Hybrid ANN-GA Model to Prediction of Bivariate Binary Responses: Application to Joint Prediction of Occurrence of Heart Block and Death in Patients with Myocardial Infarction 

**Published:** 2016-10-05

**Authors:** Negin-Sadat Mirian, Morteza Sedehi, Soleiman Kheiri, Ali Ahmadi

**Affiliations:** ^a^ Department of Biostatistics and Epidemiology, Faculty of Public Health, Shahrekord University of Medical Sciences, Shahrekord, Iran

**Keywords:** Myocardial Infarction, Heart Block, Bivariate Logistic Regression, Artificial Neural Network, Genetic Algorithm

## Abstract

**Background:** In medical studies, when the joint prediction about occurrence of two events should be anticipated, a statistical bivariate model is used. Due to the limitations of usual statistical models, other methods such as Artificial Neural Network (ANN) and hybrid models could be used. In this paper, we propose a hybrid Artificial Neural Network-Genetic Algorithm (ANN-GA) model to prediction the occurrence of heart block and death in myocardial infarction (MI) patients simultaneously.

**Methods:** For fitting and comparing the models, 263 new patients with definite diagnosis of MI hospitalized in Cardiology Ward of Hajar Hospital, Shahrekord, Iran, from March, 2014 to March, 2016 were enrolled. Occurrence of heart block and death were employed as bivariate binary outcomes. Bivariate Logistic Regression (BLR), ANN and hybrid ANN-GA models were fitted to data. Prediction accuracy was used to compare the models. The codes were written in Matlab 2013a and Zelig package in R3.2.2.

**Results:** The prediction accuracy of BLR, ANN and hybrid ANN-GA models was obtained 77.7%, 83.69% and 93.85% for the training and 78.48%, 84.81% and 96.2% for the test data, respectively. In both training and test data set, hybrid ANN-GA model had better accuracy.

**Conclusions:** ANN model could be a suitable alternative for modeling and predicting bivariate binary responses when the presuppositions of statistical models are not met in actual data. In addition, using optimization methods, such as hybrid ANN-GA model, could improve precision of ANN model.

## Introduction

The joint occurrence of events correlated with each other is always considered by researchers in medical sciences. In classical statistics, when the aim is joint prediction of two events (or response variables) somehow correlated, bivariate models are used. When both response variables are qualitative, bivariate logistic regression model is used.

Usually, traditional statistical models are based on some certain presuppositions such as specified distribution of response variables, linear relationship among dependent and independent variables, and equality of variance in errors that may not be true in actual data^1^.

Artificial Neural Network (ANN) could be an alternative method versus classical statistical models, which does not require the mentioned presuppositions of classic models and can be easily fitted for linear and nonlinear relationships^2^. Multi-layer perceptron (MLP) is the most commonly used form of ANN. Learning in MLP is done based on back-propagation (BP) algorithm by minimizing the sum of squared errors^3^.

**Figure 1:**
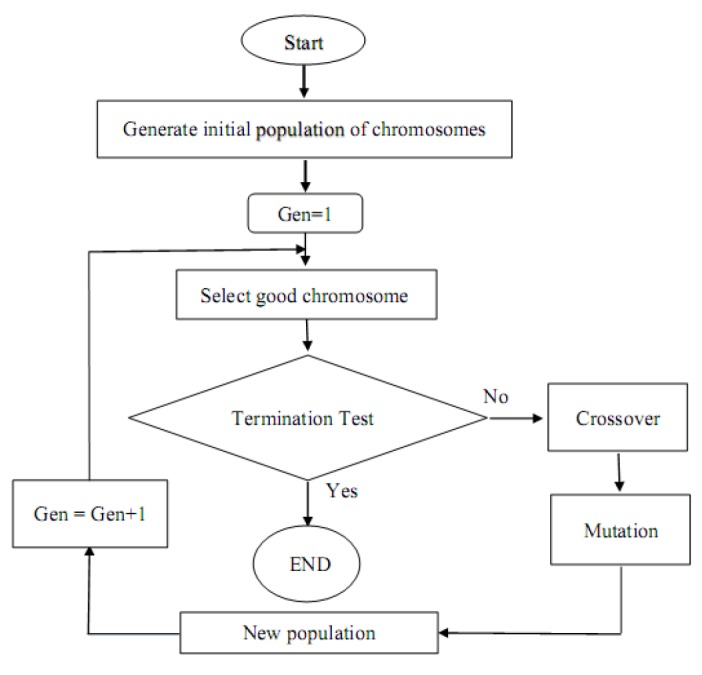
Flowchart of a typical genetic algorithm

For better efficiency of ANN model, it is necessary to optimize the parameters of model such as initial value of weights. Genetic algorithm (GA) is one of the optimization methods in ANN models^4^. GA as a technique for optimization based on Darwin theory of evolution "Survival of fittest" was first developed by John Holland. Basic operations in GA are reproduction, crossover and mutation. By combining ANN and GA, we can expect more accurate results^5^. Flowchart of a typical genetic algorithm is shown in Figure 1^6^.

Acute myocardial infarction (AMI) is referred to the constant and irrevocable cell death in a part of myocardium, which is due to the loss of blood flow and occurrence of a severe ischemia in that part. Despite wide diagnostic advancements, nearly 33% of the patients with myocardial infarction (MI) die and 5%-10% of the survived patients die within the first year after MI. There are approximately 1,500,000 AMI patients and about 25% of the mortality is attributed to this disease in USA^7^. Iran Ministry of Health and Medical Education has reported that about 39% of total mortality in Iran is due to cardiovascular diseases^8^, ^9^. Over 60% of the deaths due to MI are happen within one hour after MI and most of them are caused by arrhythmias, with ventricular fibrillation and bundle branch block as two prevalent types. Nowadays, MI is the most common cause of death in many communities and is associated in hospitals with several complications such as atrioventricular node block and bundle branch block. According to WHO report, AMI is the leading cause of mortality in the world, particularly Iran cardiac arrhythmias are the most prevalent reason for death from AMI^10^. Heart blocks are an important class of arrhythmias and lead to prolonged hospitalization and increased in-hospital mortality. Therefore, they attract attention^10^.

Because medical studies are related to human health, therefore, precise and accurate predictions are of great importance in these studies. Due to the limitations of traditional statistical methods in modeling bivariate responses, in this paper, we made an attempt to introduce a new approach with fewer restrictions based on a hybrid ANN-GA method to modeling and predicting bivariate binary responses and using this model to prediction of occurrence of heart block and death in MI patients simultaneously. We also compared prediction accuracy of this model with BLR and ANN models.

## Methods

To evaluate the suitability of the proposed model compare with traditional methods for modeling and predicting bivariate binary responses, we used data from a cross-sectional study. In this study, 263 new patients with definite diagnosis of MI hospitalized in Cardiology Ward of Hajar Hospital, Shahrekord, Iran, from March, 2014 to March, 2016 were enrolled. The diagnosis of MI was done according to the WHO criteria by a cardiologist per International Classification of Diseases (ICD10: the codes I24.9, I25.2, I22, and I21). Demographic characteristics and clinical history of the patients were gathered by a checklist at the time of admission.

In BLR model, for i-th observation, two dependent variables Y_i1_ and Y_i2_ defined that has four potential outcomes, (Y_i1_=1 ,Y_i2_=1), (Y_i1_=0 ,Y_i2_=1), (Y_i1_=1 ,Y_i2_=0), (Y_i1_=0 ,Y_i2_=0) ^11^. The joint probability π_rs_=Pr(Y_1_=r, Y_2_=s) is modeled with marginal probability π_1_=Pr(Y_1_=1) and π_2_=Pr(Y_2_=1), and ψ, which parameterizes dependence between dependent variables. The model defined as:

Y_11_‏~Bernoulli(y_11_|π_11_)

Y_10_‏~Bernoulliy_10_|π_10_

Y_01‏_~Bernoulliy_01_|π_01_ and

$$\psi =\frac{\pi_{00}.\pi_{01}}{\pi_{10}.\pi_{11}}$$

Where π_00_=1-π_11_-π_10_-π_01_.Thus for each observation: $\pi_{j}=\frac{1}{1+\exp(-x_{j}\beta_{j})}j=1,2\;,\psi =\exp(x_{3}\beta_{3})$, and joint probabilities are calculated as follows:

**Figure:**
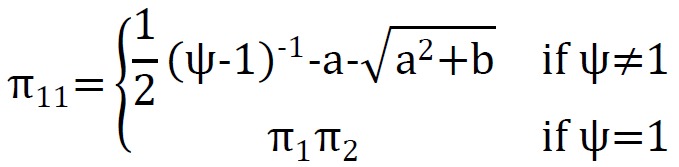

π10=π1-π11,

π01=π2-π11,

π00=1-π10-π01-π11

Where a=1+(π_1_+π_2_)(ψ-1) and b=-4ψ(ψ-1)π_1_π_2_^12,13^. For fitting BLR model, gender, the type of MI, history of diabetes, history of hypertension, dyslipidemias, history of heart disease, the rate of cardiac output fraction, systolic blood pressure, diastolic blood pressure, fasting blood sugar , non-fasting blood sugar, cholesterol, triglyceride, low-density blood cholesterol, smoking and the level of troponin enzyme considered as independent (input) variables, and occurrence of heart block (y_1_) as well as occurrence of death (y_2_) during hospitalization, employed as two dependent binary variables (outcomes). We used 184 (70%) cases as training data set and 79 (30%) cases as test data set. Model was fitted with the training data set. Test data set is used for assessment of validity of model (cross validation).

For fitting of ANN model, the training and test data set were used as with the bivariate logistic regression. Since, in this research, the outcome is bivariate, so, assuming p input nodes, where p is the number of covariates, 1 hidden layer, M nodes in hidden layer and 2 nodes in output layer, the ANN architecture can be written as:

$$y_{ik}=\psi_{0}\left(\beta_{0k}+\sum_{j=1}^{M}\beta_{jk}\psi_{h}\left(w_{j0}+\sum_{s=1}^{p}x_{is}w_{is}\right)\right)\;1,....,n\;k=1,2$$

where w_js_ is the weight for input x_is_ at the hidden node j. Also, β_j_ is the weight dependent to the hidden node j, and w_j0_ and β_0_ are the biases for the hidden and the output nodes respectively. The function Ψ_h_ is activation functions of hidden layer and the function Ψ_o_ is activation functions of output layer^2^.

We fitted MLP with one hidden layer, including 8-14 nodes. To identify the number of nodes in hidden layer, mean square error (MSE) criterion was used. Sigmoid activation function was considered for hidden and output layers. Several training algorithms including gradient descent (GD), gradient descent momentum (GDM), conjugate gradient algorithm (CGA), scaled conjugate gradient (SCG), Broyden-Fletcher-Goldfarb-Shanno (BFGS), one step secant (OSS) and Levenbery-Marqwardt (LM) were used for training. All these algorithms are from BP algorithm family^14^.

**Figure 2:**
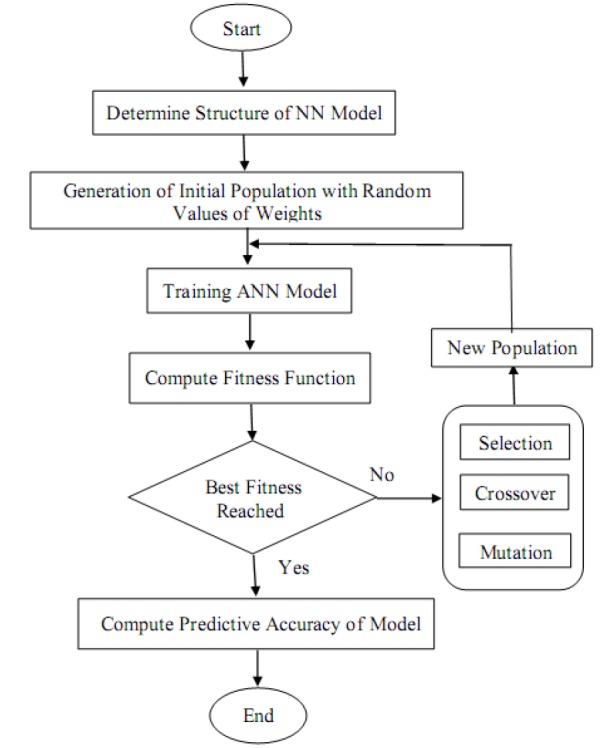
Flowchart of proposed hybrid ANN-GA model

After determining the final architecture of ANN model and select the best training algorithm, genetic algorithm was used optimize initial weights in ANN model and hybrid ANN-GA model was fitted to data. Figure 2 shows the stages of implementation of proposed hybrid model to optimize the initial values of the weights in ANN by genetic algorithm. The prediction in the bivariate models was considered correct, when both *y*_1_ and *y*_2_ variables are predicted correctly by models. Prediction accuracy was used for evaluating the models. This criterion was defined as percentage of correct joint prediction of the two binary outcomes. To implement the models, Matlab 2013a for ANN and ANN-GA models and Zelig package in R3.2.2 for bivariate logistic regression model were used^13^.

## Results

Of the 263 samples, 221 people (84.0%) had experienced heart block that (6.3%) of them died and 42 people (15.9%) had not experienced heart block that (19.0%) of them died. Correlation between two outcome variables was significant (*P*=0.006). Tables 1 and 2 present the descriptive information of general characteristics of patients.

**Table 1:** General characteristics of quantitative variables for myocardial infarction patients

**Variables**	**With heart block**		**Without heart block**		***P*** ** value**	**With death**		**Without death**		***P*** ** value**
	**Mean**	**SD**	**Mean**	**SD**		**Mean**	**SD**	**Mean**	**SD**	
Age (yr)	67.19	1.80	60.33	0.91	0.002	60.59	3.43	61.50	0.85	0.761
Level of troponin (ng/mL)	9.40	2.22	13.03	1.89	0.412	38.45	12.69	10.08	1.31	0.001
Rate of cardiac output fraction	35.29	10.40	40.90	7.52	0.001	33.91	10.63	40.56	7.83	0.001
Systolic blood pressure (mmHg)	136.79	29.12	132.01	24.25	0.251	127.27	29.75	133.28	24.63	0.283
Diastolic blood pressure (mmHg)	78.52	22.51	78.46	19.01	0.980	76.68	22.50	78.81	19.29	0.342
Fasting blood sugar(mg/dL)	177.07	82.59	147.80	66.84	0.013	200.45	85.58	148.09	67.27	0.001
Non-fasting blood sugar (mg/dL)	28.60	26.22	27.29	26.92	0.770	26.36	22.35	27.61	27.17	0.830
Cholesterol (mg/dL)	202.38	61.89	202.7	63.32	0.970	223.09	74.71	200.20	61.62	0.103
Triglyceride (mg/dL)	39.55	31.87	37.42	29.77	0.520	33.95	26.51	38.11	30.39	0.532
High-density lipid (mg/dL)	43.79	10.47	46.52	27.53	0.520	42.36	13.32	46.42	26.42	0.472

**Table 2:** General characteristics of qualitative variables for myocardial infarction patients

**Variables**	**With heart Block**		**Without heart Block**		***P*** ** value**	**With death**		**Without death**		***P*** ** value**
		**Number**	**Percent**	**Number**		**Percent**	**Number**	**Percent**	**Number**		**Percent**
Gender (Male)	30	71.4	167	75.6	0.571	16	72.7	181	75.1	0.806
History of diabetes (yes)	31	73.8	172	77.8	0.569	17	77.3	186	77.2	0.992
History of hypertension (yes)	23	54.8	138	62.4	0.349	12	54.5	149	61.8	0.502
Dyslipidemias (yes)	32	76.2	171	77.4	0.167	17	77.3	186	77.2	0.992
History of Heart Diseases (yes)	20	47.6	159	71.9	0.002	12	54.5	167	69.3	0.156
Smoking (yes)	14	33.3	110	49.8	0.050	8	36.4	116	48.1	0.290

The results of the bivariate logistic regression model for significant independent variables are shown in Table 3. Age, level of troponin and history of heart disease were significant variables in bivariate model. Prediction accuracy of ANN model with different training algorithms for training and test data set is presented in Table 4. Among different training algorithms in ANN model, LM algorithm had the highest performance.

**Table 3:** Results of bivariate logistic regression model for significant independent variables

**Variables**	**Coefficient**	**SE of Coefficient**	***P*** ** value**
Intercept (1)	-3.87	1.16	0.001
Intercept (2)	-8.85	2.02	0.001
Intercept (3)	1.84	0.71	0.011
Age (yr)	0.07	0.02	0.002
Level of Troponin	0.02	0.07	0.006
History of heart disease	1.05	0.44	0.010

**Table 4:** Prediction accuracy of different training algorithms in Artificial Neural Network (ANN) model

**Training algorithm**	**GDA**	**CGA**	**GDM**	**OSS**	**SCG**	**BFGS**	**LM**
Training Data set	78.80	79.34	77.17	83.15	79.34	78.48	83.69
Test Data set	81.01	81.00	79.70	83.54	79.74	76.63	84.81

GD: gradient descent algorithm; CGA: conjugate gradient algorithm; GDM: gradient descent momentum; OSS: one step secant; SCG: scaled conjugate gradient; BFGS: Broyden-Fletcher-Goldfarb-Shanno; LM: Levenbery-Marqwardt

Table 5 compares prediction accuracy of hybrid ANN-GA model against BLR and ANN models. In both training and test data set, hybrid ANN-GA model had better accuracy compared with other models.

**Table 5:** Prediction accuracy of models for training and test data set

**Model**	**BLR**	**ANN (LM)**	** Hybrid ANN-GA**
Training Data Set	77.70	83.69	93.85
Test Data Set	78.48	84.81	96.20

BLR: bivariate logistic regression; ANN: artificial neural network (with LM algorithm); Hybrid ANN-GA: hybrid artificial neural network-genetic algorithm

## Discussion

In this paper, we proposed a new approach based on a hybrid ANN-GA model to joint prediction of bivariate dependent binary outcomes. We compared prediction accuracy of this model with other traditional models for joint prediction of occurrence of heart block and death in MI patients. Results showed that proposed hybrid ANN-GA model had better performance compared with BLR and ANN models. Better performance of ANN model compared to classic models has been confirmed already^14-16^. Because the ANN model lacks many of limitations of classic models, in many situations, it can be a suitable alternative for these models when some (or all) of their conditions are not met in the analysis of actual data^15^. Besides, results of this study showed that hybrid ANN-GA model, because of optimization of parameters of ANN model, can improve precision of ANN model.

Despite the benefits of ANN and hybrid ANN-GA models, these methods suffer from some limitations and problems. For example, in these models, statistical inference for parameters and checking significant relationship between dependent and independent variables are not possible, because, the distribution of the parameters is not specified in ANN and hybrid models^15^.

ANN and hybrid models are more appropriate when priority is prediction of dependent variables, or data have a nonlinear and complex structure. If the primary aim is to explain a clear association among dependent and independent variables and to study the effect of independent variables on dependent variables, then classic models such as logistic regression model is preferable^17^.

Given the limitations of conventional statistical methods for modeling bivariate responses in actual data, using the proposed method in the present study is also recommended for similar problems.

## Conclusions

Hybrid ANN-GA model is the best for prediction of heart block and death simultaneously in MI patients compared with ANN and BLR models, so, considering the importance of accurate prediction in medical studies and due to the limitations of classical statistical methods for modeling bivariate responses, the use of NN and hybrid ANN-GA models is a suitable alternative for analysis of bivariate binary responses.

## Acknowledgments

The present study was extracted from MSc thesis and was supported by a grant number 1968 from the Research and Technology Deputy of Shahrekord University of Medical Sciences.

## Conflict of interest statement

None declared.
